# Metabolomics profiling reveals novel markers for leukocyte telomere length

**DOI:** 10.18632/aging.100874

**Published:** 2016-01-20

**Authors:** Jonas Zierer, Gabi Kastenmüller, Karsten Suhre, Christian Gieger, Veryan Codd, Pei-Chien Tsai, Jordana Bell, Annette Peters, Konstantin Strauch, Holger Schulz, Stephan Weidinger, Robert P. Mohney, Nilesh J. Samani, Tim Spector, Massimo Mangino, Cristina Menni

**Affiliations:** ^1^ Department of Twin Research & Genetic Epidemiology, King's College London, London, UK; ^2^ Institute of Bioinformatics and Systems Biology, Helmholtz Zentrum München, Neuherberg, Germany; ^3^ Department of Physiology and Biophysics, Weill Cornell Medical College in Qatar, Doha, Qatar; ^4^ Research Unit of Molecular Epidemiology, Helmholtz Zentrum München, German Research Center for Environmental Health, Neuherberg, Germany; ^5^ Institute of Epidemiologie II, Helmholtz Zentrum München, German Research Center for Environmental Health, Neuherberg, Germany; ^6^ German Center for Diabetes Research (DZD e.V.), Neuherberg, Germany; ^7^ Department of Cardiovascular Sciences, University of Leicester, Leicester, UK; ^8^ Institute of Genetic Epidemiology, Helmholtz Zentrum München, Neuherberg, Germany; ^9^ Institute of Epidemiology I, Helmholtz Zentrum München, Neuherberg, Germany; ^10^ Comprehensive Pneumology Center Munich (CPC-M), Member of the German Center for Lung Research, Munich, Germany; ^11^ Department of Dermatology, Venereology and Allergy, University Hospital Schleswig-Holstein, Campus Kiel, Kiel, Germany; ^12^ Metabolon, Inc. Durham, NC 27713, USA; ^13^ National Institute for Health Research (NIHR) Leicester Cardiovascular Biomedical Research Unit, Glenfield Hospital, Leicester, UK; ^14^ National Institute for Health Research (NIHR) Biomedical Research Centre at Guy's and St. Thomas' Foundation Trust, London, UK

**Keywords:** telomere length, biological aging, metabolomics, glutathione, oxidative stress

## Abstract

Leukocyte telomere length (LTL) is considered one of the most predictive markers of biological aging. The aim of this study was to identify novel pathways regulating LTL using a metabolomics approach. To this end, we tested associations between 280 blood metabolites and LTL in 3511 females from TwinsUK and replicated our results in the KORA cohort. We furthermore tested significant metabolites for associations with several aging-related phenotypes, gene expression markers and epigenetic markers to investigate potential underlying pathways. Five metabolites were associated with LTL: Two lysolipids, 1-stearoylglycerophosphoinositol (P=1.6×10^−5^) and 1-palmitoylglycerophosphoinositol (P=1.6×10^−5^), were found to be negatively associated with LTL and positively associated with phospholipase A2 expression levels suggesting an involvement of fatty acid metabolism and particularly membrane composition in biological aging. Moreover, two gamma-glutamyl amino acids, gamma-glutamyltyrosine (P=2.5×10^−6^) and gamma-glutamylphenylalanine (P=1.7×10^−5^), were negatively correlated with LTL. Both are products of the glutathione cycle and markers for increased oxidative stress. Metabolites were also correlated with functional measures of aging, i.e. higher blood pressure and HDL cholesterol levels and poorer lung, liver and kidney function. Our results suggest an involvement of altered fatty acid metabolism and increased oxidative stress in human biological aging, reflected by LTL and age-related phenotypes of vital organ systems.

## INTRODUCTION

Telomeres are repetitive DNA sequences located at the end of each chromatid. Several proteins, such as the telomere repeat-binding factor (TRF) 1 and 2, bind specifically to this area forming large nucleoprotein complexes, the t-loops [1]. These structures protect the DNA from degradation and end-to-end fusion. Telomeres shorten with each cell cycle, due to the inability of the DNA polymerase to replicate the end of the lagging strand. Thus, the shortening of telomeres has been proposed as a “mitotic clock” which limits the replicative life span of cells and causes cell senescence [2]. In fact, leucocyte telomere length (LTL) has been associated not only with chronological age [3] but also many aging-related diseases, such as Alzheimer's Disease (AD) [4,5], cardiovascular disease [6,7] and cancer [8,9]. Furthermore, LTL was found to predict mortality [10,11] and longevity [12]. Thus, it was suggested as potential biomarker of biological aging [13].

Genome-wide association studies have until now identified ten genes associated with LTL [14,15,16,17]. Most of these genes physically interact with telomeres; however, how the shortening of telomeres affects an individual's health is still not fully understood. Recent developments in the field of metabolomics allow for the high-throughput measurement of an extensive set of low-molecular-weight molecules (metabolites) [18]. Changes in metabolite concentrations reflect physiological functions and can indicate early stages of diseases [19]. Recently, a study on LTL revealed strong associations with blood biomarkers in a cohort of American Indians [20]. However, the study was small (n=423) and lacked independent replication.

In this study, we assess to which extent metabolic profiles are correlated with LTL in a large population study (n=3511, females only) from the UK using a non-targeted metabolomics platform. We replicate our results in an independent cohort from Germany (n=904). Furthermore, we examine the relationship of the LTL-associated metabolites with aging-related phenotypes as well as gene expression and methylation markers in order to gain insights in the mechanisms of biological aging.

## RESULTS

The demographic characteristics of the study populations are presented in Table 1. We analyzed the associations between 280 fasting blood metabolites and LTL in 3511 women from the TwinsUK cohort (see supplemental Table 1).

**Table 1:** Population Characteristics

	TwinsUK	KORA
*N*	3511	904
*Age (yrs)*	53.6 ± 13.6	60.5 ± 8.8
MZ:DZ:Singletons*	1654:1360:497	0:0:904
*TL*	3.72 ± 0.67	1.85 ± 0.31
*BMI (kg/m^2^)*	26.21 ± 5.14	27.87 ± 5.25
*FEV1 (l)*	2.60 ± 0.61	2.79 ± 0.50
*HDL (mmol/L)*	1.71 ± 0.48	
*DBP (mm Hg)*	78.01 ± 10.68	
*SBP (mm Hg)*	126.71 ± 18.20	
*ALAT (IU/L)*	27.63 ± 17.07	
*GGT (U/L)*	28.36 ± 25.44	
*eGFR (mL/min/1.73m^2^)*	83.78 ± 17.07	
*smoking (non:ex:current)*	1905:1134:447	

* MZ=monozygotic, DZ=dizygotic

We found two lipids (1-stearoylglycerophosphoinositol: Beta [95%CI] =−0.07 [−0.10:−0.04] change in metabolite z-score per change in LTL z-score, P=1.6×10^−5^ and 1-palmitoylglycerophosphoinositol: Beta [95%CI] =−0.08 [−0.12:−0.04], P=1.6×10^−5^), two gamma-glutamyl-amino acids (gamma-glutamyltyrosine: Beta [95%CI] =−0.08 [−0.11:−0.05], P=2.5×10^−6^ and gamma-glutamylphenyl-alanine: Beta [95%CI] =−0.07 [−0.10:−0.04], P=1.7×10^−5^), and one xenobiotic (4-vinylphenol sulfate: Beta [95%CI] =−0.07 [−0.10:−0.03], P=1.4×10^−4^) to be negatively associated with LTL after adjustment for potential confounding factors and after correcting the results for multiple testing (Table 2, Supplemental Figure 1). All five metabolites showed the same effects with similar effect sizes in 904 female individuals from the KORA F4 study, even though they did not reach significance level. All metabolites remained Bonferroni-significant (P<1.8×10^−4^) after meta-analysis.

**Table 2:** Metabolites significantly associated with LTL

		TwinsUK		KORA	Meta	
Metabolite	PW	beta [95%CI]	p	beta [95%CI]	beta [95%CI]	p
gamma-glutamyltyrosine	Peptide	−0.09 [−0.12:−0.05]	3.41×10^−6^	−0.05 [−0.12:0.02]	−0.08 [−0.11:−0.05]	2.51×10^−6^
1-stearoylglycero-phosphoinositol	Lipid	−0.09 [−0.13:−0.05]	1.36×10^−6^	−0.00 [−0.07:0.07]	−0.07 [−0.10:−0.04]	1.60×10^−5^
1-palmitoylglycero-phosphoinositol	Lipid	−0.08 [−0.13:−0.04]	7.36×10^−5^	−0.07 [−0.14:0.01]	−0.08 [−0.12:−0.04]	1.64×10^−5^
gamma-glutamyl-phenylalanine	Peptide	−0.08 [−0.12:−0.04]	2.72×10^−5^	−0.04 [−0.11:0.02]	−0.07 [−0.10:−0.04]	1.68×10^−5^
4-vinylphenol sulfate	Xenobiotic	−0.08 [−0.12:−0.04]	7.41×10^−5^	−0.03 [−0.10:0.05]	−0.07 [−0.10:−0.03]	1.41×10^−4^

Three multivariate Lasso models were fitted to predict LTL: The first using clinical variables only (age, BMI), the second using the five identified metabolites only, and the third using both clinical variables and metabolites. The model based on metabolites alone could not achieve the performance of the model based on clinical variables alone, however, combining clinical variables with metabolites significantly improved the prediction in the combined model (Figure 2). In the combined model, 1-stearoylglycerophosphoinisitol was the strongest predictor followed by the 4-vinylphenol sulfate. All five metabolites were selected in the optimal Lasso model (beta < 0), suggesting non-redundant associations with LTL. The coefficient of determination, a measure of goodness of fit, of the final model was estimated at 14.5% in a leave-one-out validation.

**Figure 1:**
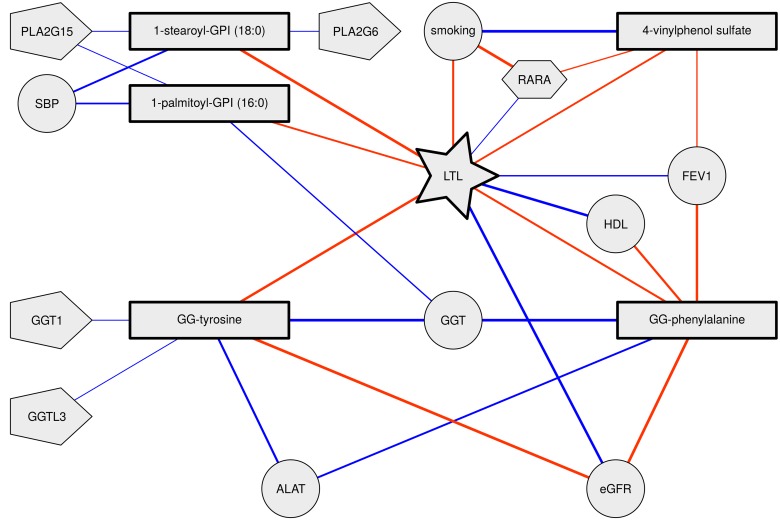
Telomere length, metabolite and phenotype interrelationships Nodes represent variables where rectangles represent metabolites, circles represent phenotypes, pentagons represent expression levels and hexagons represent DNA methylation levels. Links between nodes represent significant correlations (red negative, blue positive). Thicker edges indicate stronger correlations.

**Figure 2:**
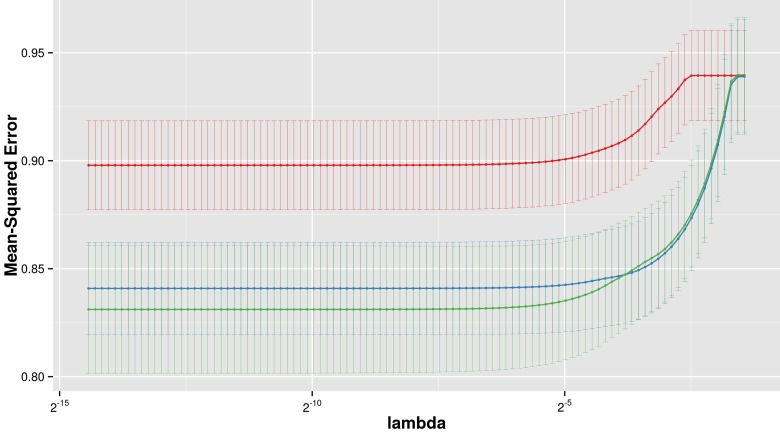
LTL prediction performance The figure shows the prediction performance (mean square error on Y axis) of three different Lasso models, based on metabolites only (red), clinical variables only (blue) and metabolites with clinical variables combined (green), dependent on the amount of regularization (lambda on x axis).

Moreover, we found all five metabolites to be strongly associated with several aging-related phenotypes independently of chronological age (Table 3): Both lysolipids correlated with increased systolic blood pressure (1-stearoylglycerophosphoinositol: Beta=1.09 [0.56:1.61], P=5.3×10^−5^ and 1-palmitoylglycero-phosphoinositol: Beta=1.10 [0.52:1.67], P=1.7×10^−4^). Additionally, 1-palmitoyl-glycerophosphoinositol was found to be associated with the serum concentration of gamma-glutamyl transpeptidase (GGT), a measure of liver function (Beta=0.08 [0.03:0.12], P=1.0×10^−3^).

**Table 3:** Phenotypes associated with LTL and associated metabolites

	phenotype	beta [95%CI]	p
telomere length	HDL cholesterol	0.04 [0.02:0.06]	2.50×10^−6^
	eGFR	1.42 [0.82:2.01]	2.79×10^−6^
	smoking	−0.06 [−0.08:−0.03]	3.17×10^−5^
	FEV1	0.03 [0.01:0.05]	8.85×10^−4^
1-palmitoylglycerophosphoinositol	SBP	1.10 [0.52:1.67]	1.76×10^−4^
	GGT	0.08 [0.03:0.12]	1.04×10^−3^
1-stearoylglycerophosphoinositol	SBP	1.09 [0.56:1.61]	5.34×10^−5^
4-vinylphenol sulfate	smoking	0.24 [0.22:0.26]	2.32×10^−102^
	FEV1	−0.02 [−0.04:−0.01]	1.40×10^−3^
gamma-glutamylphenylalanine	eGFR	−2.24 [−2.74:−1.73]	3.14×10^−18^
	GGT	0.15 [0.10:0.19]	3.21×10^−12^
	FEV1	−0.03 [−0.05:−0.02]	4.48×10^−6^
	HDL cholesterol	−0.03 [−0.05:−0.02]	1.15×10^−5^
	ALAT	0.10 [0.05:0.14]	5.76×10^−5^
gamma-glutamyltyrosine	GGT	0.14 [0.10:0.19]	5.41×10^−11^
	eGFR	−1.65 [−2.19:−1.11]	1.58×10^−9^
	ALAT	0.11 [0.06:0.16]	1.67×10^−5^

The two gamma-glutamyl amino acids were strongly associated with the estimated glomerular filtration rate (eGFR), a marker for renal function (gamma-glutamyltyrosine: Beta=−1.65 [−2.19:−1.11], P=1.6×10^−9^ and gamma-glutamylphenylalanine: Beta=−2.24 [−2.74:−1.73], P=3.1×10^−18^), and two markers of liver function, namely GGT and alanine amino transaminase (ALAT) (GGT: Beta=0.14 [0.10:0.19], P=5.4×10^−11^ and Beta=0.15 [0.10:0.19], P=3.2×10^−12^ respectively; ALAT: Beta=0.11 [0.06:0.16], P=1.7×10^−5^ and Beta=0.10 [0.05:0.14], P=5.8×10^−5^ respectively). Gamma-glutamylphenylalanine was additionally associated with lung function, measured as forced expiratory volume in one second (FEV1, Beta=−0.03 [−0.05:−0.02], P=4.5×10^−6^), and HDL cholesterol levels (Beta=−0.03 [−0.05:−0.02], P=1.1×10^−5^).

Moreover, the xenobiotic 4-vinylphenol sulfate was strongly associated with tobacco smoking (Beta=0.24 [0.22:0.26], P=2.3×10^−102^) and also weakly with FEV1 (Beta=−0.02 [−0.04:−0.01], P=1.4×10^−3^).

Thus, all five metabolites were consistently associated with accelerated biological aging, i.e. shorter telomeres, higher blood pressure and higher HDL cholesterol levels and poorer lung, liver and kidney function (Table 3 and Figure 1).

To further investigate mechanisms of biological aging, we analyzed the association of the five significant metabolites with gene expression levels of related enzymes, namely GGT and phospholipase A2 (PLA2), in a subset of 753 individuals with RNA chip data from available LCL probes available. We found gamma-glutamyltyrosine was positively associated with GGT1 and GGTL3 gene expressions (probes ILMN_2274240: Beta=0.09 [0.02:0.15], P=0.01 and ILMN_1786186: Beta=0.07 [0.00:0.14], P=0.04). Also, 1-stearoylglycerophosphoinositol was positively associated with expression of the PLA2 gene PLA2G15 (probe ILMN_1756910: Beta=0.09 [0.01:0.16], P=0.02 and probe ILMN_1798955: Beta=0.08 [0.00:0.15], P=0.05) as well as 1-palmitoylglycerophosphoinositol (probe ILMN_1756910: Beta=0.08 [0.00:0.16], P=0.05).

The metabolite 4-vinylphenol sulfate is known to be associated with several DNA methylation probes, possibly driven by tobacco smoking [21,22]. We found one of these probes, cg19572487, being significantly associated with LTL (Beta= 0.10 [0.04:0.17], P=9×10^−3^), smoking (Beta=−0.51 [−0.63:−0.39], P=9×10^−16^) and 4-vinylphenol sulfate levels (Beta=−0.05 [−0.09:−0.02], P=1×10^−3^) in our data. The probe is located on chromosome 17 in the retinoic acid receptor, alpha (RARA) gene.

## DISCUSSION

In the largest study of this kind, we searched for molecular markers and mechanisms involved in LTL regulation using a metabolomics approach. We identified five novel blood metabolites, namely gamma-glutamyltyrosine, gamma-glutamylphenylalanine, 1-stearoylglycerophosphoinositol, 1-palmitoylglycero-phosphoinositol and 4-vinylphenol sulfate, independently associated with LTL with high statistical significance. These metabolites belong to three different classes: lysolipids, gamma-glutamyl amino acids and xenobiotics, which will be discussed in the following.

### Lysolipids

Lysolipids are produced from glycerophospholipids by the enzyme phospholipase A2 (PLA2), which releases one of the fatty acids from the glycerol backbone [23]. Glycerophospholipids were previously found to be positively correlated with LTL [20] while in our study, circulating levels of the lysolipids 1-stearoylglycero-phosphoinositol and 1-palmitoylglycerophosphoinositol were significantly associated with shortening of LTL. This suggests an increased activity of PLA2 in advanced biological aging. This hypothesis is further confirmed by the positive association of the two lysolipids with PLA2 gene expression levels in LCLs in our study. PLA2 activity, amongst others, affects the composition and physiology of cell membranes by catalyzing the hydrolysis of membrane lipids [24,25]. The integrity of cell membranes and their ability to resist oxidative stress have been shown to be key aspects of biological aging [26]. Studies comparing centenarians with younger controls identified alterations of cell membrane composition [27] and particularly depletion of the lysolipid stearoylphosphatidylcholine [28] as possible reasons for longevity.

Another regulator of membrane fluidity is the saturation of fatty acids. Both stearic acid and palmitic acid are saturated fatty acids that are known to decrease membrane fluidity, which in turn was associated with increased susceptibility to disease [29,30,31]. In contrast higher levels of polyunsaturated fatty acid-containing phospholipids were observed in centenarians compared to elderly [32], suggesting their involvement in retarded biological aging. These alterations of membrane composition with biological aging provide a possible explanation for previously reported association of LTL with e.g. AD [4].

### Gamma-glutamyl amino acids

We found two gamma-glutamyl amino acids, gamma-glutamyltyrosine and gamma-glutamylphenylalanine, were negatively associated with LTL. These metabolites are components of the gamma-glutamyl cycle and are produced by the degradation of glutathione (GSH) and its conjugates catalyzed by the enzyme GGT. The main purpose of this reaction is regeneration of the intracellular GSH pool, i.e. to break-down extra-cellular GSH conjugates to make its components available for reimport into the cell [33,34,35]. GSH is crucial for detoxification of reactive oxygen species (ROS) as well as other toxic compounds [33,34,35]. Thus, increased GGT activity was proposed as a marker for increased oxidative stress [33,36]. Gamma-glutamyltyrosine and gamma-glutamylphenylalanine were both highly correlated with the abundance of the GGT enzyme, as well as GGT1 and GGTL3 gene expression in this study. The serum concentration of GGT is a common clinical marker for liver function [37]. While the liver produces most of the GSH [34], in the body GGT is most active in kidneys, which absorb GSH for detoxification [34,37]. Accordingly, we also found kidney function, measured as eGFR, being highly correlated with both, LTL and gamma-glutamyl amino acids. In conclusion, the gamma-glutamyl amino acids indicate an involvement of increased oxidative stress and worsened liver and kidney function in biological aging.

We also found gamma-glutamylphenylalanine being associated with worsened lung function in both cohorts. This might also be due to oxidative stress, which was previously associated with chronic lung disease [38].

### 4-Vinylphenol sulfate

4-vinylphenol sulfate is a xenobiotic that was reported to be strongly associated with tobacco smoking [39]. We observed the same correlation in our data. Moreover, we found both 4-vinylphenol sulfate as well as LTL to be strongly correlated with cotinine abundance, which is a well-established marker for tobacco smoking. Accordingly, higher levels of 4-vinylphenol sulfate were associated with worsened lung function. Moreover, analysis of DNA methylation data from our cohort confirmed previously published associations of 4-vinylphenol sulfate with the methylation level of a CpG site in the RARA gene [21] and revealed an association of the same site with LTL and smoking. RARA is a transcription factor that was shown to regulate differentiation and apoptosis [40]. However, despite the strong correlation between LTL and smoking, we did not find a significant difference in LTL between monozygotic twins, discordant for smoking. These associations show how smoking accelerates biological aging mediated by changes in metabolism as well as DNA methylation. Smoking was shown to have a profound effect on the GSH metabolism of the lung [38], suggesting increased oxidative stress as a possible link between smoking, metabolism and LTL.

While we were able to identify five novel markers of LTL, our study has some limits. First, we analyzed data of females only and some of the identified metabolites are known to show gender-specific blood levels [41]. However, in a small pilot (n=372) from the TwinsUK cohort we observed concordant correlations between LTL and gamma-glutamyl amino acids as well as 4-vinylphenol sulfate for men as for women. In contrast, we did not see an association between any of the lysolipids and LTL in men, suggesting gender-specific changes of fatty acid metabolism with aging. Second, we did not reach statistical significance in the replication cohort. This can be attributed to smaller sample size. The power to detect the observed effects at a significance level of 0.05 in 900 individuals is only around 50%. Nonetheless, despite the lack of power, the much higher age and the different geographical location and genetic background of the replication cohort, all of the five metabolites remain Bonferroni-significant after meta-analysis.

Our results suggest two mechanisms of biological aging: On the one hand, changes in lipid metabolism and resulting changes of the cell membrane composition appear to be related to LTL and biological aging. On the other hand, we observed metabolites indicating increased oxidative stress due to alterations in the GSH metabolism, which has been previously related to LTL and aging phenotypes. One possible cause for increased oxidative stress is tobacco smoking, which might mediate the association of 4-vinylphenol sulfate with LTL. Moreover we found LTL and the related metabolites being associated with impairment of liver and kidney function. This highlights the importance of detoxification, particularly of reactive oxygen species, in biological aging.

## METHODS

### Discovery population

Study subjects were twins enrolled in the TwinsUK registry, a national register of adult twins recruited as volunteers without selecting for any particular disease or trait [42]. In this study we analyzed data from 3511 female twins who had who had complete data for LTL and metabolomics profiling.

The study was approved by St. Thomas' Hospital Research Ethics Committee, and all twins provided informed written consent.

### Replication cohort

KORA F4 is a population cohort based in the region of Augsburg, Germany [43]. The replication set consisted of 904 female individuals with serum metabolite levels, measures of telomeres and measures of lung function [44] available.

### LTL measurement

A detailed description of LTL measurement in both TwinsUK and KORA was previously described in Codd et al. [15]. In brief, mean LTL of the samples was measured using a quantitative PCR–based technique [14,45] and expressed as a ratio of telomere repeat length (T) to a copy number of a single copy gene (S). A calibrator sample or a standard curve was used for to standardize T/S results across plates. LTLs measures were inverse normalized in both cohorts.

### Metabolomics measurement

Metabolomics data was measured by Metabolon Inc., Durham, USA as previously described [46]. Briefly, metabolite concentrations were measured in blood samples using an untargeted GC/MS and LC/MS platform. Measurements were scaled by run-day median and inverse normalized in both cohorts.

### Aging phenotypes

Lung function was measured as forced expiratory volume in one second (FEV1) in line with ATS/ERS recommendations [44,47]. Furthermore, diastolic and systolic blood pressure (DBP and SBP), body mass index (BMI) and serum HDL cholesterol levels were measured during clinical visits of the study participants. Renal function was measured by estimating glomerular filtration rate (eGFR) from serum creatinine levels using the CKD-EPI equation [48]. Liver function was assessed by measuring serum gamma-glutamyl transpeptidase (GGT) and alanine amino transaminase (ALAT) concentrations. Both measures were inverse normalized prior to analysis.

### Gene expression

RNA abundance was measured in LCLs of 778 female individuals from the TwinsUK cohort using the Illumina Human HT-12 V3 BeadChip as part of the MuTHER project as previously described [49]. We selected 30 probes from GGT and PLA2 genes. Probes were adjusted for batch effects by linear models and residuals were inverse normalized prior to analysis.

### DNA methylation

DNA was extracted from whole blood, bisulfite converted and subsequently analyzed using the Infinium 450K kit as previously described [50]. The beta mixture quantile dilation (BMIQ) method was performed to correct for technical variation [51]. Measurements were inverse normalized and then adjusted for batch effects, family structure and cell counts (PlasmaBlast, CD8+CD28−CD45RA− T cells, naive CD8 T cells, CD4+ T cells, Natural Killer cells, monocytes, and granulocytes) using linear models.

### Statistical analysis

All analyses were performed using R (version 3.1.2) using the lme4 (version 1.1) package.

Correlations between metabolites and LTL were calculated using linear mixed models, correcting for age, BMI and family relatedness (as random intercept). A conservative multiple test-corrected threshold of P<1.8×10^−4^ was used to identify significant associations; this value represented P = 0.05 divided by the total number of tests performed (280 metabolites). We replicated the five Bonferroni-significant metabolites in the KORA F4 cohort. The data was consistently normalized in both cohorts. The results were meta-analyzed using inverse variance fixed effect meta-analys-is implemented in the R package meta (version 4.3).

We estimated the power of the replication cohort using the R package pwr (version 1.1), which implements power estimation according to Cohen [52].

To identify redundant associations of the metabolites, we fitted a multivariate Lasso model [53] incorporating all Bonferroni significant metabolites together with age and BMI. The predictive performance of the model was then compared to a similar model containing age and BMI only. The model performance was assessed by calculating the predicted residual sum of squares (PRESS) and subsequent P^2^ statistics using a leave-one-out cross validation.

Subsequently, we aimed to further explore the relationship of LTL and the identified metabolites with biological aging. To this end, we used linear mixed models to test for association of the previously identified metabolites with previously described aging phenotypes. All models were adjusted for age, BMI and family relatedness. The lung function parameter FEV1 was additionally adjusted for height, as suggested in the literature. We replicated the associations with lung function parameters in KORA, adjusting for the same covariates.

## SUPPLEMENTAL DATA


