# Supplementary material for: Metabolomics profiling reveals novel markers for leukocyte telomere length
Source: Aging (Albany NY). 2016 Jan 20;8(1):77–86. doi: 10.18632/aging.100874 (PMC4761715; doi:10.18632/aging.100874)
Supplement: Supplementary file 1 [file aging-08-077-s001.pdf]

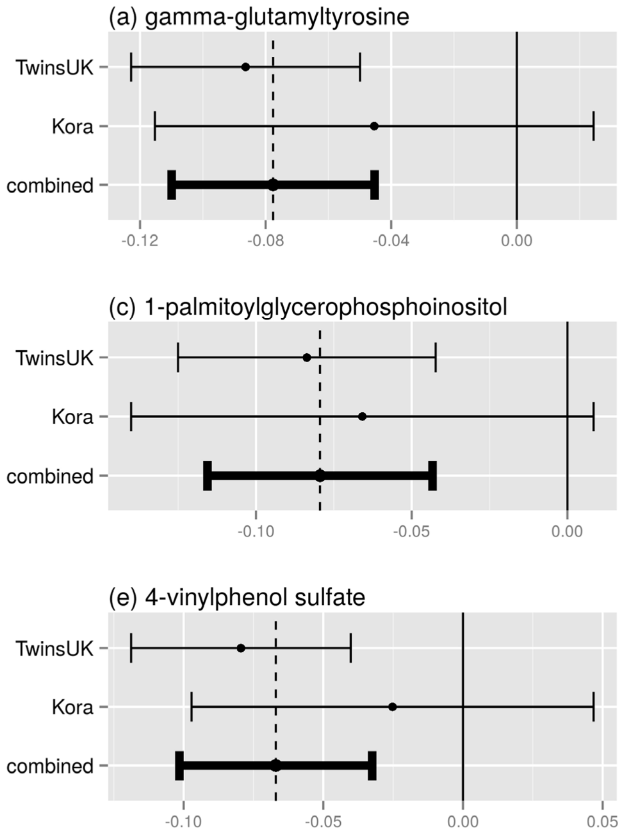


**Supplemental Figure 1. Forest plots of significant metabolites.** The forest plots show effects and confidence intervals for all significant metabolites in the discovery cohort (TwinsUK), the replication cohort (Kora) and the combined meta-analysis. The dashed lines indicate the combined effect after meta-analysis.

**Supplemental Table 1. Associations of blood metabolite levels with LTL. The table contains the complete list of all analyzed metabolites and their association with LTL. (** indicates FWER<0.05, * indicates FDR <0.05)**

| metabolite | pathway | beta [95%CI] | p |
|---|---|---|---|
| 1-stearoylglycerophosphoinositol | Lipid | -0.09 [-0.13:-0.05] | $1.36\times10^{-6}$** |
| gamma-glutamyltyrosine | Peptide | -0.09 [-0.12:-0.05] | $3.41\times10^{-6}$** |
| gamma-glutamylphenylalanine | Peptide | -0.08 [-0.12:-0.04] | $2.72\times10^{-5}$** |
| 1-palmitoylglycerophosphoinositol | Lipid | -0.08 [-0.13:-0.04] | $7.36\times10^{-5}$** |
| 4-vinylphenol sulfate | Xenobiotics | -0.08 [-0.12:-0.04] | $7.41\times10^{-5}$** |
| 1-arachidonoylglycerophosphoethanolamine | Lipid | -0.07 [-0.11:-0.03] | $2.42\times10^{-4}$* |
| 1-arachidonoylglycerophosphoinositol | Lipid | -0.07 [-0.11:-0.03] | $2.76\times10^{-4}$* |
| 1-oleoylglycerophosphoethanolamine | Lipid | -0.07 [-0.10:-0.03] | $7.60\times10^{-4}$* |
| uridine | Nucleotide | 0.06 [0.03:0.10] | $1.07\times10^{-3}$* |
| caprylate (8:0) | Lipid | -0.06 [-0.10:-0.02] | $1.42\times10^{-3}$* |
| erythritol | Xenobiotics | -0.06 [-0.09:-0.02] | $1.54\times10^{-3}$* |
| arabinose | Carbohydrate | -0.07 [-0.11:-0.02] | $1.75\times10^{-3}$* |
| ornithine | Amino acid | -0.06 [-0.10:-0.02] | $2.14\times10^{-3}$* |
| heptanoate (7:0) | Lipid | -0.06 [-0.09:-0.02] | $3.25\times10^{-3}$ |
| pyroglutamine | Amino acid | -0.05 [-0.09:-0.02] | $3.54\times10^{-3}$ |
| tyrosine | Amino acid | -0.05 [-0.09:-0.02] | $5.27\times10^{-3}$ |
| laurylcarnitine | Lipid | -0.07 [-0.11:-0.02] | $6.70\times10^{-3}$ |
| proline | Amino acid | -0.05 [-0.09:-0.01] | $7.56\times10^{-3}$ |
| malate | Energy | -0.05 [-0.09:-0.01] | $7.66\times10^{-3}$ |
| caproate (6:0) | Lipid | -0.05 [-0.09:-0.01] | $7.66\times10^{-3}$ |
| kynurenine | Amino acid | -0.05 [-0.08:-0.01] | $1.04\times10^{-2}$ |
| pseudouridine | Nucleotide | -0.05 [-0.08:-0.01] | $1.30\times10^{-2}$ |
| 1-palmitoylglycerophosphoethanolamine | Lipid | -0.05 [-0.08:-0.01] | $1.31\times10^{-2}$ |
| arachidonate (20:4n6) | Lipid | -0.05 [-0.09:-0.01] | $1.33\times10^{-2}$ |
| 3-dehydrocarnitine | Lipid | -0.05 [-0.08:-0.01] | $1.41\times10^{-2}$ |
| C-glycosyltryptophan | Amino acid | -0.04 [-0.08:-0.01] | $1.41\times10^{-2}$ |
| 1,3,7-trimethylurate | Xenobiotics | -0.06 [-0.11:-0.01] | $1.43\times10^{-2}$ |
| 4-methyl-2-oxopentanoate | Amino acid | 0.05 [0.01:0.08] | $1.56\times10^{-2}$ |
| bilirubin (E,E) | Cofactors and vitamins | 0.05 [0.01:0.08] | $1.58\times10^{-2}$ |
| p-acetamidophenylglucuronide | Xenobiotics | -0.09 [-0.17:-0.02] | $1.59\times10^{-2}$ |
| glycerol | Lipid | -0.05 [-0.08:-0.01] | $1.65\times10^{-2}$ |
| 1-eicosatrienoylglycerophosphocholine | Lipid | -0.05 [-0.08:-0.01] | $1.72\times10^{-2}$ |
| lactate | Carbohydrate | -0.05 [-0.08:-0.01] | $1.78\times10^{-2}$ |
| thromboxane B2 | Lipid | -0.04 [-0.08:-0.01] | $2.25\times10^{-2}$ |
| 1-palmitoylglycerol (1-monopalmitin) | Lipid | -0.04 [-0.08:-0.01] | $2.34\times10^{-2}$ |
| butyrylcarnitine | Lipid | -0.04 [-0.08:-0.01] | $2.37\times10^{-2}$ |
| 2-hydroxyglutarate | Lipid | -0.05 [-0.10:-0.01] | $2.38\times10^{-2}$ |
| homocitrulline | Amino acid | -0.06 [-0.12:-0.01] | $2.74\times10^{-2}$ |
| phenylalanine | Amino acid | -0.04 [-0.08:-0.00] | $2.74\times10^{-2}$ |
| alpha-hydroxyisovalerate | Amino acid | -0.04 [-0.08:-0.00] | $3.01\times10^{-2}$ |
| CMPF | Lipid | 0.04 [0.00:0.07] | $3.16\times10^{-2}$ |

| | | | |
|---|---|---|---|
| biliverdin | Cofactors and vitamins | 0.04 [0.00:0.08] | $3.47\times10^{-2}$ |
| cholesterol | Lipid | -0.04 [-0.07:-0.00] | $3.64\times10^{-2}$ |
| oleoylcarnitine | Lipid | 0.04 [0.00:0.08] | $3.83\times10^{-2}$ |
| pelargonate (9:0) | Lipid | -0.04 [-0.07:-0.00] | $4.00\times10^{-2}$ |
| paraxanthine | Xenobiotics | -0.04 [-0.08:-0.00] | $4.15\times10^{-2}$ |
| 3-(cystein-S-yl)acetaminophen | Xenobiotics | -0.09 [-0.18:-0.00] | $4.15\times10^{-2}$ |
| urate | Nucleotide | -0.04 [-0.07:-0.00] | $4.18\times10^{-2}$ |
| carnitine | Lipid | -0.04 [-0.08:-0.00] | $4.35\times10^{-2}$ |
| 1-linoleoylglycerophosphoethanolamine | Lipid | -0.04 [-0.08:-0.00] | $4.44\times10^{-2}$ |
| 1-stearoylglycerophosphoethanolamine | Lipid | -0.04 [-0.08:-0.00] | $4.46\times10^{-2}$ |
| adrenate (22:4n6) | Lipid | -0.04 [-0.08:-0.00] | $4.49\times10^{-2}$ |
| gamma-glutamylvaline | Peptide | -0.04 [-0.07:-0.00] | $4.83\times10^{-2}$ |
| threonate | Cofactors and vitamins | 0.04 [0.00:0.08] | $4.96\times10^{-2}$ |
| tetradecanedioate | Lipid | -0.04 [-0.08:0.00] | $5.11\times10^{-2}$ |
| hyodeoxycholate | Lipid | -0.04 [-0.08:0.00] | $5.11\times10^{-2}$ |
| 2-hydroxystearate | Lipid | -0.04 [-0.07:0.00] | $5.18\times10^{-2}$ |
| ibuprofen | Xenobiotics | -0.07 [-0.13:0.00] | $5.21\times10^{-2}$ |
| 2-methoxyacetaminophen sulfate | Xenobiotics | -0.08 [-0.17:0.00] | $5.22\times10^{-2}$ |
| 2-hydroxypalmitate | Lipid | -0.04 [-0.07:0.00] | $5.24\times10^{-2}$ |
| hexadecanedioate | Lipid | -0.04 [-0.08:0.00] | $5.36\times10^{-2}$ |
| gamma-glutamylthreonine | Peptide | -0.06 [-0.11:0.00] | $5.55\times10^{-2}$ |
| alanine | Amino acid | -0.04 [-0.07:0.00] | $5.73\times10^{-2}$ |
| dihomo-linolenate (20:3n3 or n6) | Lipid | -0.04 [-0.07:0.00] | $5.88\times10^{-2}$ |
| undecanoate (11:0) | Lipid | -0.04 [-0.07:0.00] | $6.04\times10^{-2}$ |
| glutamate | Amino acid | -0.03 [-0.07:0.00] | $6.14\times10^{-2}$ |
| glycerate | Carbohydrate | 0.03 [-0.00:0.07] | $7.26\times10^{-2}$ |
| ADpSGEGDFXAEGGGVR | Peptide | 0.06 [-0.01:0.12] | $7.39\times10^{-2}$ |
| naproxen | Xenobiotics | -0.33 [-0.68:0.02] | $8.20\times10^{-2}$ |
| saccharin | Xenobiotics | -0.06 [-0.12:0.01] | $8.32\times10^{-2}$ |
| aspartylphenylalanine | Peptide | -0.05 [-0.12:0.01] | $8.33\times10^{-2}$ |
| beta-hydroxyisovalerate | Amino acid | -0.03 [-0.07:0.00] | $8.51\times10^{-2}$ |
| propionylcarnitine | Lipid | -0.03 [-0.07:0.00] | $8.61\times10^{-2}$ |
| 7-Hoca | Lipid | -0.03 [-0.07:0.00] | $8.69\times10^{-2}$ |
| cyclo(leu-pro) | Peptide | -0.04 [-0.09:0.01] | $8.73\times10^{-2}$ |
| estrone 3-sulfate | Lipid | -0.09 [-0.18:0.01] | $8.92\times10^{-2}$ |
| benzoate | Xenobiotics | -0.03 [-0.07:0.01] | $9.32\times10^{-2}$ |
| 1-methylxanthine | Xenobiotics | -0.04 [-0.08:0.01] | $9.34\times10^{-2}$ |
| N-acetylalanine | Amino acid | -0.03 [-0.07:0.01] | $9.61\times10^{-2}$ |
| pyridoxate | Cofactors and vitamins | 0.03 [-0.01:0.07] | $9.70\times10^{-2}$ |
| 4-acetamidobutanoate | Amino acid | -0.03 [-0.07:0.01] | $9.80\times10^{-2}$ |
| gamma-glutamylleucine | Peptide | -0.03 [-0.07:0.01] | $1.01\times10^{-1}$ |
| 4-androsten-3beta,17beta-diol disulfate 1 | Lipid | -0.03 [-0.07:0.01] | $1.07\times10^{-1}$ |
| 1-arachidonoylglycerophosphocholine | Lipid | -0.03 [-0.07:0.01] | $1.07\times10^{-1}$ |
| 1-palmitoylplasmenylethanolamine | Lipid | -0.03 [-0.08:0.01] | $1.07\times10^{-1}$ |
| 2-hydroxyisobutyrate | Amino acid | 0.03 [-0.01:0.07] | $1.11\times10^{-1}$ |

| | | | |
|---|---|---|---|
| caffeine | Xenobiotics | -0.03 [-0.07:0.01] | $1.15\times10^{-1}$ |
| 3-methyl-2-oxovalerate | Amino acid | 0.03 [-0.01:0.07] | $1.20\times10^{-1}$ |
| 4-acetamidophenol | Xenobiotics | -0.08 [-0.18:0.02] | $1.23\times10^{-1}$ |
| N2,N2-dimethylguanosine | Nucleotide | -0.03 [-0.07:0.01] | $1.34\times10^{-1}$ |
| cholate | Lipid | 0.03 [-0.01:0.08] | $1.36\times10^{-1}$ |
| succinylcarnitine | Energy | -0.03 [-0.07:0.01] | $1.38\times10^{-1}$ |
| stachydrine | Xenobiotics | 0.03 [-0.01:0.07] | $1.40\times10^{-1}$ |
| citrate | Energy | 0.03 [-0.01:0.06] | $1.42\times10^{-1}$ |
| 1-palmitoleoylglycerophosphocholine | Lipid | -0.03 [-0.07:0.01] | $1.44\times10^{-1}$ |
| gamma-glutamylisoleucine | Peptide | -0.03 [-0.07:0.01] | $1.47\times10^{-1}$ |
| phenyllactate | Amino acid | -0.03 [-0.07:0.01] | $1.51\times10^{-1}$ |
| hydroxyisovaleroyl carnitine | Amino acid | -0.03 [-0.08:0.01] | $1.54\times10^{-1}$ |
| isoleucine | Amino acid | -0.03 [-0.06:0.01] | $1.55\times10^{-1}$ |
| palmitate (16:0) | Lipid | -0.03 [-0.07:0.01] | $1.58\times10^{-1}$ |
| 1,7-dimethylurate | Xenobiotics | -0.03 [-0.07:0.01] | $1.59\times10^{-1}$ |
| creatinine | Amino acid | -0.03 [-0.06:0.01] | $1.61\times10^{-1}$ |
| 3-(3-hydroxyphenyl)propionate | Amino acid | 0.07 [-0.03:0.16] | $1.64\times10^{-1}$ |
| 4-hydroxyhippurate | Xenobiotics | -0.03 [-0.08:0.01] | $1.65\times10^{-1}$ |
| N-acetylornithine | Amino acid | -0.03 [-0.07:0.01] | $1.82\times10^{-1}$ |
| N-(2-furoyl)glycine | Xenobiotics | -0.10 [-0.25:0.05] | $1.87\times10^{-1}$ |
| palmitoylcarnitine | Lipid | 0.02 [-0.01:0.06] | $2.02\times10^{-1}$ |
| 1-heptadecanoylglycerophosphocholine | Lipid | 0.03 [-0.01:0.06] | $2.05\times10^{-1}$ |
| histidine | Amino acid | 0.02 [-0.01:0.06] | $2.08\times10^{-1}$ |
| 4-ethylphenylsulfate | Xenobiotics | -0.03 [-0.07:0.02] | $2.09\times10^{-1}$ |
| 3-methyl-2-oxobutyrate | Amino acid | 0.02 [-0.01:0.06] | $2.09\times10^{-1}$ |
| phenol sulfate | Amino acid | -0.02 [-0.06:0.01] | $2.11\times10^{-1}$ |
| 1,5-anhydroglucitol (1,5-AG) | Carbohydrate | -0.02 [-0.06:0.01] | $2.12\times10^{-1}$ |
| alpha-tocopherol | Cofactors and vitamins | 0.02 [-0.01:0.06] | $2.20\times10^{-1}$ |
| 2-hydroxyacetaminophen sulfate | Xenobiotics | -0.04 [-0.12:0.03] | $2.23\times10^{-1}$ |
| mannose | Carbohydrate | -0.02 [-0.06:0.01] | $2.27\times10^{-1}$ |
| glycochenodeoxycholate | Lipid | -0.02 [-0.06:0.02] | $2.36\times10^{-1}$ |
| piperine | Xenobiotics | 0.02 [-0.02:0.06] | $2.38\times10^{-1}$ |
| deoxycholate | Lipid | -0.03 [-0.08:0.02] | $2.39\times10^{-1}$ |
| nonadecanoate (19:0) | Lipid | 0.02 [-0.01:0.06] | $2.41\times10^{-1}$ |
| hippurate | Xenobiotics | 0.02 [-0.02:0.06] | $2.42\times10^{-1}$ |
| 3-indoxyl sulfate | Amino acid | -0.02 [-0.06:0.02] | $2.52\times10^{-1}$ |
| bilirubin (Z,Z) | Cofactors and vitamins | 0.02 [-0.02:0.06] | $2.55\times10^{-1}$ |
| myristate (14:0) | Lipid | -0.02 [-0.06:0.02] | $2.56\times10^{-1}$ |
| hexanoylcarnitine | Lipid | -0.02 [-0.06:0.02] | $2.61\times10^{-1}$ |
| 2-tetradecenoyl carnitine | Lipid | -0.02 [-0.06:0.02] | $2.63\times10^{-1}$ |
| myo-inositol | Lipid | -0.02 [-0.06:0.02] | $2.70\times10^{-1}$ |
| palmitoyl sphingomyelin | Lipid | -0.02 [-0.06:0.02] | $2.70\times10^{-1}$ |
| palmitoleate (16:1n7) | Lipid | -0.02 [-0.06:0.02] | $2.74\times10^{-1}$ |
| ursodeoxycholate | Lipid | -0.02 [-0.07:0.02] | $2.77\times10^{-1}$ |
| homostachydrine | Xenobiotics | -0.04 [-0.11:0.03] | $2.78\times10^{-1}$ |

| | | | |
|---|---|---|---|
| lathosterol | Lipid | -0.02 [-0.06:0.02] | $2.84\times10^{-1}$ |
| laurate (12:0) | Lipid | -0.02 [-0.06:0.02] | $2.86\times10^{-1}$ |
| 3-phenylpropionate (hydrocinnamate) | Amino acid | 0.02 [-0.02:0.06] | $2.86\times10^{-1}$ |
| glutaroyl carnitine | Amino acid | -0.02 [-0.06:0.02] | $2.88\times10^{-1}$ |
| DHA (22:6n3) | Lipid | 0.02 [-0.02:0.06] | $2.92\times10^{-1}$ |
| bradykinin, des-arg(9) | Peptide | 0.03 [-0.02:0.08] | $3.01\times10^{-1}$ |
| 7-methylxanthine | Xenobiotics | 0.02 [-0.02:0.07] | $3.05\times10^{-1}$ |
| erythrose | Carbohydrate | 0.02 [-0.02:0.06] | $3.07\times10^{-1}$ |
| epiandrosterone sulfate | Lipid | -0.02 [-0.05:0.02] | $3.13\times10^{-1}$ |
| 1-stearoylglycerol (1-monostearin) | Lipid | -0.02 [-0.06:0.02] | $3.13\times10^{-1}$ |
| 10-heptadecenoate (17:1n7) | Lipid | -0.02 [-0.06:0.02] | $3.23\times10^{-1}$ |
| glucose | Carbohydrate | -0.02 [-0.06:0.02] | $3.37\times10^{-1}$ |
| myristoleate (14:1n5) | Lipid | -0.02 [-0.06:0.02] | $3.38\times10^{-1}$ |
| 10-undecenoate (11:1n1) | Lipid | 0.02 [-0.02:0.06] | $3.41\times10^{-1}$ |
| threonine | Amino acid | -0.02 [-0.06:0.02] | $3.45\times10^{-1}$ |
| taurochenodeoxycholate | Lipid | -0.02 [-0.06:0.02] | $3.45\times10^{-1}$ |
| salicyluric glucuronide | Xenobiotics | -0.06 [-0.19:0.07] | $3.50\times10^{-1}$ |
| 5alpha-androstan-3beta,17beta-diol disulfate | Lipid | -0.02 [-0.06:0.02] | $3.54\times10^{-1}$ |
| 1-methylurate | Xenobiotics | -0.02 [-0.06:0.02] | $3.56\times10^{-1}$ |
| dodecanedioate | Lipid | -0.02 [-0.06:0.02] | $3.56\times10^{-1}$ |
| GPC | Lipid | -0.02 [-0.06:0.02] | $3.57\times10^{-1}$ |
| tryptophan | Amino acid | 0.02 [-0.02:0.06] | $3.62\times10^{-1}$ |
| 1-oleoylglycerol (1-monoolein) | Lipid | -0.02 [-0.07:0.02] | $3.64\times10^{-1}$ |
| catechol sulfate | Xenobiotics | -0.02 [-0.06:0.02] | $3.64\times10^{-1}$ |
| mannitol | Carbohydrate | -0.02 [-0.06:0.02] | $3.71\times10^{-1}$ |
| pantothenate | Cofactors and vitamins | 0.02 [-0.02:0.06] | $3.74\times10^{-1}$ |
| gamma-tocopherol | Cofactors and vitamins | -0.02 [-0.06:0.02] | $3.76\times10^{-1}$ |
| asparagine | Amino acid | 0.02 [-0.02:0.05] | $3.79\times10^{-1}$ |
| 1-stearoylglycerophosphocholine | Lipid | 0.02 [-0.02:0.05] | $3.83\times10^{-1}$ |
| lysine | Amino acid | -0.02 [-0.05:0.02] | $3.83\times10^{-1}$ |
| stearoylcarnitine | Lipid | 0.02 [-0.02:0.05] | $3.89\times10^{-1}$ |
| 2-stearoylglycerophosphocholine | Lipid | 0.02 [-0.02:0.05] | $3.93\times10^{-1}$ |
| 2-methylbutyroylcarnitine | Amino acid | -0.02 [-0.05:0.02] | $4.03\times10^{-1}$ |
| 1,6-anhydroglucose | Carbohydrate | -0.02 [-0.07:0.03] | $4.07\times10^{-1}$ |
| glycocholate | Lipid | 0.02 [-0.02:0.06] | $4.08\times10^{-1}$ |
| leucine | Amino acid | -0.02 [-0.05:0.02] | $4.10\times10^{-1}$ |
| 15-methylpalmitate (isobar with 2-methylpalmitate) | Lipid | -0.02 [-0.06:0.03] | $4.14\times10^{-1}$ |
| acetylphosphate | Energy | -0.01 [-0.05:0.02] | $4.31\times10^{-1}$ |
| 5alpha-pregnan-3beta,20alpha-diol disulfate | Lipid | 0.02 [-0.03:0.06] | $4.42\times10^{-1}$ |
| cysteine | Amino acid | -0.01 [-0.05:0.02] | $4.43\times10^{-1}$ |
| phenylacetate | Amino acid | 0.02 [-0.03:0.06] | $4.54\times10^{-1}$ |
| n-Butyl Oleate | Lipid | 0.02 [-0.03:0.07] | $4.56\times10^{-1}$ |
| cis-4-decenoyl carnitine | Lipid | -0.02 [-0.06:0.03] | $4.58\times10^{-1}$ |
| creatine | Amino acid | 0.01 [-0.02:0.05] | $4.67\times10^{-1}$ |
| 3-(4-hydroxyphenyl)lactate | Amino acid | -0.01 [-0.05:0.02] | $4.74\times10^{-1}$ |

| | | | |
|---|---|---|---|
| 7-methylguanine | Nucleotide | -0.02 [-0.06:0.03] | $4.88\times10^{-1}$ |
| citrulline | Amino acid | -0.01 [-0.05:0.02] | $4.93\times10^{-1}$ |
| DHEA-S | Lipid | -0.01 [-0.04:0.02] | $5.03\times10^{-1}$ |
| pentadecanoate (15:0) | Lipid | -0.01 [-0.05:0.03] | $5.10\times10^{-1}$ |
| 5-dodecenoate (12:1n7) | Lipid | -0.01 [-0.05:0.03] | $5.13\times10^{-1}$ |
| 4-androsten-3beta,17beta-diol disulfate 2 | Lipid | -0.01 [-0.05:0.03] | $5.15\times10^{-1}$ |
| arginine | Amino acid | 0.01 [-0.03:0.05] | $5.19\times10^{-1}$ |
| linoleate (18:2n6) | Lipid | -0.01 [-0.05:0.03] | $5.27\times10^{-1}$ |
| octanoylcarnitine | Lipid | -0.01 [-0.05:0.03] | $5.32\times10^{-1}$ |
| atenolol | Xenobiotics | -0.06 [-0.25:0.13] | $5.39\times10^{-1}$ |
| phenylalanylphenylalanine | Peptide | 0.02 [-0.04:0.08] | $5.40\times10^{-1}$ |
| tryptophan betaine | Amino acid | 0.01 [-0.03:0.05] | $5.42\times10^{-1}$ |
| androsterone sulfate | Lipid | -0.01 [-0.05:0.02] | $5.44\times10^{-1}$ |
| N-acetylthreonine | Amino acid | -0.01 [-0.05:0.03] | $5.46\times10^{-1}$ |
| 2-hydroxyhippurate (salicylurate) | Xenobiotics | 0.02 [-0.04:0.08] | $5.65\times10^{-1}$ |
| glycerol 2-phosphate | Xenobiotics | 0.01 [-0.03:0.05] | $5.90\times10^{-1}$ |
| dihomo-linoleate (20:2n6) | Lipid | -0.01 [-0.05:0.03] | $5.94\times10^{-1}$ |
| valine | Amino acid | -0.01 [-0.05:0.03] | $5.94\times10^{-1}$ |
| carbamazepine | Xenobiotics | 0.12 [-0.31:0.55] | $6.01\times10^{-1}$ |
| 3-hydroxybutyrate (BHBA) | Lipid | 0.01 [-0.03:0.05] | $6.05\times10^{-1}$ |
| N1-methyladenosine | Nucleotide | -0.01 [-0.05:0.03] | $6.16\times10^{-1}$ |
| betaine | Amino acid | 0.01 [-0.03:0.05] | $6.21\times10^{-1}$ |
| decanoylcarnitine | Lipid | 0.01 [-0.03:0.05] | $6.21\times10^{-1}$ |
| N-acetylglycine | Amino acid | 0.01 [-0.03:0.05] | $6.28\times10^{-1}$ |
| isovalerate | Lipid | -0.01 [-0.05:0.03] | $6.34\times10^{-1}$ |
| allantoin | Nucleotide | -0.01 [-0.05:0.03] | $6.40\times10^{-1}$ |
| chiro-inositol | Lipid | 0.02 [-0.05:0.08] | $6.42\times10^{-1}$ |
| salicylate | Xenobiotics | -0.01 [-0.08:0.05] | $6.47\times10^{-1}$ |
| glycine | Amino acid | 0.01 [-0.03:0.05] | $6.50\times10^{-1}$ |
| serine | Amino acid | 0.01 [-0.03:0.05] | $6.56\times10^{-1}$ |
| cotinine | Xenobiotics | 0.02 [-0.08:0.12] | $6.65\times10^{-1}$ |
| dimethylarginine (SDMA + ADMA) | Amino acid | 0.01 [-0.03:0.05] | $6.78\times10^{-1}$ |
| hypoxanthine | Nucleotide | -0.01 [-0.05:0.03] | $6.89\times10^{-1}$ |
| theobromine | Xenobiotics | -0.01 [-0.05:0.03] | $6.90\times10^{-1}$ |
| erythronate | Carbohydrate | 0.01 [-0.03:0.05] | $6.90\times10^{-1}$ |
| taurocholate | Lipid | 0.01 [-0.04:0.06] | $6.92\times10^{-1}$ |
| alpha-ketoglutarate | Energy | 0.01 [-0.03:0.05] | $7.12\times10^{-1}$ |
| stearidonate (18:4n3) | Lipid | -0.01 [-0.04:0.03] | $7.13\times10^{-1}$ |
| 2-palmitoylglycerophosphocholine | Lipid | -0.01 [-0.04:0.03] | $7.32\times10^{-1}$ |
| eicosenoate (20:1n9 or 11) | Lipid | 0.01 [-0.03:0.04] | $7.44\times10^{-1}$ |
| pyruvate | Carbohydrate | 0.01 [-0.03:0.04] | $7.54\times10^{-1}$ |
| valerate | Lipid | -0.01 [-0.06:0.04] | $7.55\times10^{-1}$ |
| fructose | Carbohydrate | 0.01 [-0.03:0.04] | $7.57\times10^{-1}$ |
| EPA | Lipid | 0.01 [-0.03:0.04] | $7.58\times10^{-1}$ |
| stearate (18:0) | Lipid | -0.01 [-0.04:0.03] | $7.61\times10^{-1}$ |

| | | | |
|---|---|---|---|
| octadecanedioate | Lipid | 0.01 [-0.03:0.04] | $7.73\times10^{-1}$ |
| urea | Amino acid | -0.01 [-0.04:0.03] | $7.75\times10^{-1}$ |
| indoleacetate | Amino acid | 0.01 [-0.03:0.04] | $7.76\times10^{-1}$ |
| cortisone | Lipid | -0.01 [-0.04:0.03] | $7.76\times10^{-1}$ |
| levulinate (4-oxovalerate) | Amino acid | 0.01 [-0.03:0.04] | $7.80\times10^{-1}$ |
| oleate (18:1n9) | Lipid | -0.01 [-0.04:0.03] | $7.82\times10^{-1}$ |
| margarate (17:0) | Lipid | -0.01 [-0.04:0.03] | $7.83\times10^{-1}$ |
| 1-oleoylglycerophosphocholine | Lipid | 0.01 [-0.03:0.04] | $7.87\times10^{-1}$ |
| taurolithocholate 3-sulfate | Lipid | -0.00 [-0.05:0.04] | $8.10\times10^{-1}$ |
| leucylleucine | Peptide | -0.01 [-0.06:0.05] | $8.21\times10^{-1}$ |
| ADSGEGDFXAEGGGVR | Peptide | 0.01 [-0.04:0.05] | $8.23\times10^{-1}$ |
| 3-methoxytyrosine | Amino acid | -0.00 [-0.05:0.04] | $8.26\times10^{-1}$ |
| 2-hydroxybutyrate (AHB) | Amino acid | 0.00 [-0.03:0.04] | $8.29\times10^{-1}$ |
| methionine | Amino acid | 0.00 [-0.03:0.04] | $8.31\times10^{-1}$ |
| pipecolate | Amino acid | -0.00 [-0.04:0.03] | $8.33\times10^{-1}$ |
| 2-oleoylglycerophosphocholine | Lipid | 0.00 [-0.03:0.04] | $8.36\times10^{-1}$ |
| 1-linoleoylglycerophosphocholine | Lipid | 0.00 [-0.03:0.04] | $8.40\times10^{-1}$ |
| scyllo-inositol | Lipid | 0.00 [-0.04:0.05] | $8.42\times10^{-1}$ |
| glutamine | Amino acid | -0.00 [-0.04:0.03] | $8.44\times10^{-1}$ |
| indolelactate | Amino acid | -0.00 [-0.04:0.03] | $8.47\times10^{-1}$ |
| heme | Cofactors and vitamins | 0.00 [-0.04:0.05] | $8.56\times10^{-1}$ |
| 2-aminobutyrate | Amino acid | 0.00 [-0.03:0.04] | $8.58\times10^{-1}$ |
| 10-nonadecenoate (19:1n9) | Lipid | -0.00 [-0.04:0.03] | $8.78\times10^{-1}$ |
| 1-myristoylglycerophosphocholine | Lipid | -0.00 [-0.04:0.03] | $8.80\times10^{-1}$ |
| thymol sulfate | Xenobiotics | -0.00 [-0.06:0.05] | $8.81\times10^{-1}$ |
| pro-hydroxy-pro | Peptide | 0.00 [-0.04:0.04] | $8.82\times10^{-1}$ |
| trans-4-hydroxyproline | Amino acid | 0.00 [-0.04:0.04] | $8.86\times10^{-1}$ |
| cortisol | Lipid | 0.00 [-0.04:0.04] | $8.87\times10^{-1}$ |
| 5-oxoproline | Amino acid | 0.00 [-0.04:0.04] | $8.91\times10^{-1}$ |
| threitol | Carbohydrate | -0.00 [-0.04:0.03] | $8.91\times10^{-1}$ |
| G3P | Lipid | 0.00 [-0.03:0.04] | $9.01\times10^{-1}$ |
| serotonin | Amino acid | -0.00 [-0.05:0.04] | $9.04\times10^{-1}$ |
| acetylcarnitine | Lipid | -0.00 [-0.04:0.04] | $9.04\times10^{-1}$ |
| isobutyrylcarnitine | Amino acid | 0.00 [-0.04:0.04] | $9.05\times10^{-1}$ |
| HWESASXX | Peptide | -0.00 [-0.04:0.03] | $9.10\times10^{-1}$ |
| bilirubin (E,Z or Z,E) | Cofactors and vitamins | 0.00 [-0.04:0.05] | $9.15\times10^{-1}$ |
| metoprolol acid metabolite | Xenobiotics | -0.01 [-0.19:0.18] | $9.24\times10^{-1}$ |
| 1-docosahexaenoylglycerophosphocholine | Lipid | 0.00 [-0.04:0.04] | $9.37\times10^{-1}$ |
| ergothioneine | Xenobiotics | -0.00 [-0.05:0.05] | $9.38\times10^{-1}$ |
| p-cresol sulfate | Amino acid | 0.00 [-0.04:0.04] | $9.41\times10^{-1}$ |
| indolepropionate | Amino acid | 0.00 [-0.04:0.04] | $9.42\times10^{-1}$ |
| choline | Lipid | 0.00 [-0.04:0.04] | $9.43\times10^{-1}$ |
| isovalerylcarnitine | Amino acid | -0.00 [-0.04:0.04] | $9.45\times10^{-1}$ |
| 2-linoleoylglycerophosphocholine | Lipid | -0.00 [-0.04:0.04] | $9.46\times10^{-1}$ |
| docosapentaenoate (n3 DPA; 22:5n3) | Lipid | 0.00 [-0.04:0.04] | $9.47\times10^{-1}$ |

| | | | |
|---|---|---|---|
| 1-palmitoylglycerophosphocholine | Lipid | -0.00 [-0.04:0.04] | $9.50\times10^{-1}$ |
| phosphate | Energy | -0.00 [-0.04:0.04] | $9.53\times10^{-1}$ |
| phenylacetylglutamine | Amino acid | 0.00 [-0.04:0.04] | $9.60\times10^{-1}$ |
| 3-methylxanthine | Xenobiotics | 0.00 [-0.04:0.05] | $9.61\times10^{-1}$ |
| DSGEGDFXAEGGGVR | Peptide | -0.00 [-0.05:0.05] | $9.62\times10^{-1}$ |
| xanthine | Nucleotide | -0.00 [-0.04:0.04] | $9.80\times10^{-1}$ |
| gamma-glutamylglutamine | Peptide | -0.00 [-0.04:0.04] | $9.99\times10^{-1}$ |
